# CASBench: A Benchmarking Set of Proteins with Annotated Catalytic and Allosteric Sites in Their Structures

**Published:** 2019

**Authors:** A. Zlobin, D. Suplatov, K. Kopylov, V. Švedas

**Affiliations:** Lomonosov Moscow State University, Belozersky Institute of Physicochemical Biology and Faculty of Bioengineering and Bioinformatics, Lenin hills 1, bldg. 73, 119991, Moscow, Russia

**Keywords:** ligand binding sites, catalytic site, allosteric site, benchmarking set, protein function and regulation, structure-function relationship, bioinformatics, web server

## Abstract

In recent years, the phenomenon of allostery has witnessed growing attention driven by a fundamental interest in new ways to regulate the functional properties of proteins, as well as the prospects of using allosteric sites as targets to design novel drugs with lower toxicity due to a higher selectivity of binding and specificity of the mechanism of action. The currently available bioinformatic methods can sometimes correctly detect previously unknown ligand binding sites in protein structures. However, the development of universal and more efficient approaches requires a deeper understanding of the common and distinctive features of the structural organization of both functional (catalytic) and allosteric sites, the evolution of their amino acid sequences in respective protein families, and allosteric communication pathways. The CASBench benchmark set contains 91 entries related to enzymes with both catalytic and allosteric sites within their structures annotated based on the experimental information from the Allosteric Database, Catalytic Site Atlas, and Protein Data Bank. The obtained dataset can be used to benchmark the performance of existing computational approaches and develop/train perspective algorithms to search for new catalytic and regulatory sites, as well as to study the mechanisms of protein regulation on a large collection of allosteric enzymes. Establishing a relationship between the structure, function, and regulation is expected to improve our understanding of the mechanisms of action of enzymes and open up new prospects for discovering new drugs and designing more efficient biocatalysts. The CASBench can be operated offline on a local computer or online using built-in interactive tools at https://biokinet.belozersky.msu.ru/casbench.

## INTRODUCTION

Allostery is a mechanism by which the activity of proteins is regulated due to binding of a ligand or other protein in a special site on the surface [1]. Fifty years ago, when the classical models for cooperativity binding were proposed based on the first known cases of allostery, this phenomenon was considered an exclusive feature of multi-subunit proteins functioning at the level of the quaternary structure [2, 3]. Recent studies have provided a large body of evidence for allostery in proteins having different structures and functions, including small monomeric proteins. It has become clear that allostery is not an exclusive property of sophisticated multi-subunit complexes, but rather a widespread phenomenon that plays a key role in the regulation of many biological processes [4-8]. Computational biology methods were applied to study allostery in an attempt to understand the relationship between function and regulation [6, 9]. Bioinformatic analysis showed that the amino acid sequence of regulatory sites is less conserved and more variable compared to that in catalytic sites [10]. The catalytic and allosteric sites are saturated by the so-called specific positions that are conserved only within functional subfamilies but differ between them and can define the functional diversity of homologs within one superfamily (e.g., they can be responsible for varying specificity to substrates and regulatory ligands) [11, 12, 13].

Analysis of correlated substitutions in the amino acid sequences of topologically independent but functionally coupled sites on the surface of evolutionary related proteins is increasingly being used to study the molecular mechanisms of allostery [9, 14]. It was shown that such correlating/co-evolving positions can form a network of interacting residues located between the catalytic and regulatory sites in a protein structure, which provides communication between them due to the sequential conformational changes initiated by the binding of a regulatory agent [15, 16]. Co-evolution between positions located in different binding sites at a considerable distance from each other was described (e.g., in the bacterial transcription factors belonging to the LacI family [17]). Spatially proximal co-evolving residue pairs, as well as long-range correlations, can potentially be used to annotate new binding sites and study the molecular mechanisms of allosteric communication [18].

The current understanding is that the protein structure exists in equilibrium between a set of conformers, and this balance can change as a result of the binding of almost any substance to the surface of the globule; the question is only how efficient this shift is and how it affects the protein function [19-22]. This effectively means that allostery can present a universal phenomenon that applies to the majority of existing proteins. The anticipation of the discovery of a new regulatory mechanism in proteins currently considered as non-allosteric has generated intense attention to the field, driven by a fundamental interest in establishing new ways of regulating proteins/ enzymes, and the prospects for creating novel allosteric drugs having a lower toxicity due to higher binding selectivity [4, 23-26]. In recent years, a number of computational methods have been developed to search for new regulatory sites in protein structures, as well as complementary selective ligands that can influence the functional activity upon binding to the biopolymer [9]: using geometric [27-30], energy-based [31, 32] or bioinformatic criteria [13, 33, 34, 35], training sets of experimentally annotated sites [36, 37], and high-throughput virtual screening procedures [38, 39]. The currently available computer programs usually predict multiple sites in the structure of a selected protein (tens or even hundreds, depending on the globule size and the selected parameters). The functional importance of the detected pockets is then estimated by ranking them according to a chosen evaluation function (e.g., according to the occurrence of statistically significant conserved [34, 35] or specific positions [13]). The currently available bioinformatic methods can sometimes correctly detect previously unknown ligand binding sites in protein structures, but it is clear that the efficiency of the available software for allosteric site prediction remains very limited and that new universal computational approaches are needed to proceed from individual case studies towards a wider solution to this problem. We can also note the common limitation of the known search algorithms: they do not take into account the differences between the functional (catalytic) and regulatory (allosteric) sites and, therefore, are unable to discriminate between sites of different types. To elaborate more efficient strategies, it is necessary to systematically study the common patterns, as well as the distinctive features of structural organization of sites of different types and the evolution of their amino acid sequences in families of homologous proteins.

The first attempt to summarize the accumulated experimental information on allosteric sites was the ASD database, which contains almost two thousand entries [40]. The ASD database is an important resource on allosteric proteins but contains redundant (duplicated) data and low-quality annotations; so, only a small part of this collection can be used in practice to study allostery and train/evaluate new algorithms (235 entries [41]). In addition, annotation of functional (catalytic) sites is not provided in the ASD database: this information is presented in the separate CSA database [42]. The CSA database relies on experimental findings for one thousand enzymes. The bioinformatics methods are used to annotate conserved catalytic residues in the closest homologs with a known structure, thus expanding the database to dozens of thousands of entries. The combined use of experimental information on known catalytic and allosteric sites in the structures of proteins/enzymes can help in the study of the relationship between the structure, function, and regulation, but certain issues regarding data management and the format of entries in the ASD and CSA databases make their joint use a challenging task.

Here, we report on the CASBench set of enzymes with catalytic and allosteric binding sites, with their structures annotated according to the experimental data in the ASD, CSA, and PDB public databases. The CASBench can be used as a benchmarking set to evaluate the efficiency of existing methods and to develop new, promising algorithms to search for functional and regulatory sites in protein structures. The availability of annotations for both sites in each protein provides an opportunity to study the organization of sites of different types and to train computer algorithms to recognize them. The CASBench can be operated offline on a local computer or online using built-in interactive tools.

## METHODS

**Collection of the CASBench set**

The latest versions of three public databases (annotations of allosteric sites in the ASD, annotations of catalytic sites in the CSA, and structural information contained in the PDB database) were analyzed by the original Python 3 software using the BioPython package [43], as well as the numpy and ProDy libraries. The protocol employed to collect the CASBench dataset contained four key steps: (1) numbering of allosteric site residues in the ASD was synchronized with the numbering of amino acid residues in the corresponding representative PDB structures; (2) for each protein in the ASD, all its structures in the PDB were retrieved; (3) the ASD entries were compared to the CSA entries to identify proteins deposited in both databases; and (4) annotations of catalytic and allosteric sites in the ASD and CSA databases were refined using information about the presence of ligands in all collected PDB structures of crystallographic complexes and taking into account the quaternary structure of each protein (when available).

At the first step, annotations of allosteric sites in the ASD were synchronized with the corresponding PDB entries regarding the numbering of amino acid residues. The primary difficulty in working with the ASD is the ambiguous numbering of amino acid residues that are part of a regulatory site; i.e., it may fail to match the numbering in the PDB and/or Uniprot, and can even be represented by only a keyword (e.g., DFG motif), which in some cases prevents conclusive identification of the site in a protein structure. If the numbering of amino acid residues in the ASD annotation failed to match the numbering in the PDB or UniProt, it was automatically corrected by considering all possible locations of the amino acid residues of the site in question in the sequence of each corresponding PDB chain, with allowance for potential substitutions, deletions, or insertions. All entries in the ASD whose automatic synchronization had failed (i.e., it was not possible to conclusively identify the allosteric site in the PDB structure given the ASD numbering) were removed from analysis. At the second step, for each protein in the ASD, all of its structures currently available in the PDB were collected. The amino acid sequences of all proteins presented in the PDB were clustered at a 95% sequence similarity level using the CD-HIT program [44] (i.e., the PDB95 set). All members of a cluster that contained a representative PDB-structure were included in the corresponding ASD entry for further analysis. The quaternary structure of each protein (if any) was restored according to the corresponding BIOMT records. At the third stage, the ASD and CSA databases were compared. Annotations of catalytic sites in CSA for the proteins with at least 95% sequence similarity were merged into one entry. Proteins that were present in the ASD but not in the CSA (i.e., none of the PDB structures retrieved at the previous stage was annotated in the CSA) were excluded from further consideration. At the final step of the protocol, the primary annotations of catalytic and allosteric sites in proteins were refined as follows: Sites in the ASD and CSA databases can be represented by only a few residues whose role in function or regulation has been confirmed experimentally (e.g., the key catalytically important amino acids studied by site-directed mutagenesis), which gives no information about the dimensions and boundaries of the corresponding binding sites. All the available experimental information from the crystallographic complexes of proteins with ligands was used to refine these primary annotations. For each protein present in both the ASD and CSA databases, the collected information on all its structures in the PDB was used to select ligands bound to corresponding sites within 5 Å of any amino acid residue included in the primary annotation. Then, this primary annotation of catalytic and allosteric sites from the ASD and CSA databases was supplemented with the secondary annotation obtained by analyzing the available crystallographic complexes. In each structure, all residues located within 5 Å of the selected ligand were considered and the resulting secondary annotations of each site were merged for all the PDB structures of the protein. The collected CASBench set contained 91 enzymes.

**Construction of multiple alignments of protein families**

Unique chains of each protein in the CASBench were used as queries to construct multiple alignments of the corresponding families using the Mustguseal method [45]. For each query, protein sequence similarity search versus the UniProtKB database was used to collect at most 5,000 homologs for further analysis [46]. The resulting sets were filtered to exclude the too similar and too distant proteins. Sequences were dismissed if their length differed by more than 20% from the query to exclude incomplete entries and prevent the formation of columns with an excess of gaps in the final alignment. The CD-HIT algorithm [44] was then used to cluster proteins at a sequence similarity threshold of 95%. One representative sequence was automatically selected from each cluster, and the remaining proteins were dismissed from further consideration. Finally, proteins that differed significantly in their amino acid sequence from the query (shared less than 0.25 bits per column) and, therefore, could have caused align ment errors were removed [47, 48]. Multiple sequence alignments of the obtained representative collections of each family were finally constructed using the MAFFT algorithm [49].

## RESULTS AND DISCUSSION

**Fig. 1:**
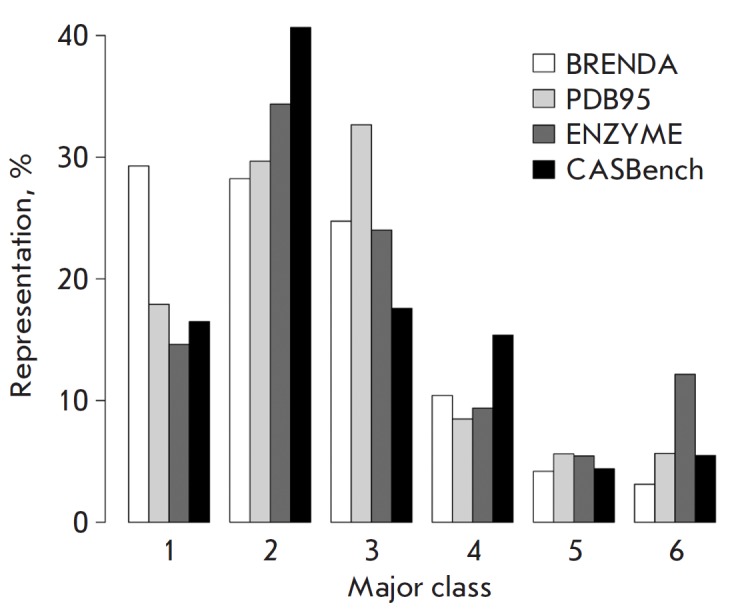
The ...

**Fig. 2:**
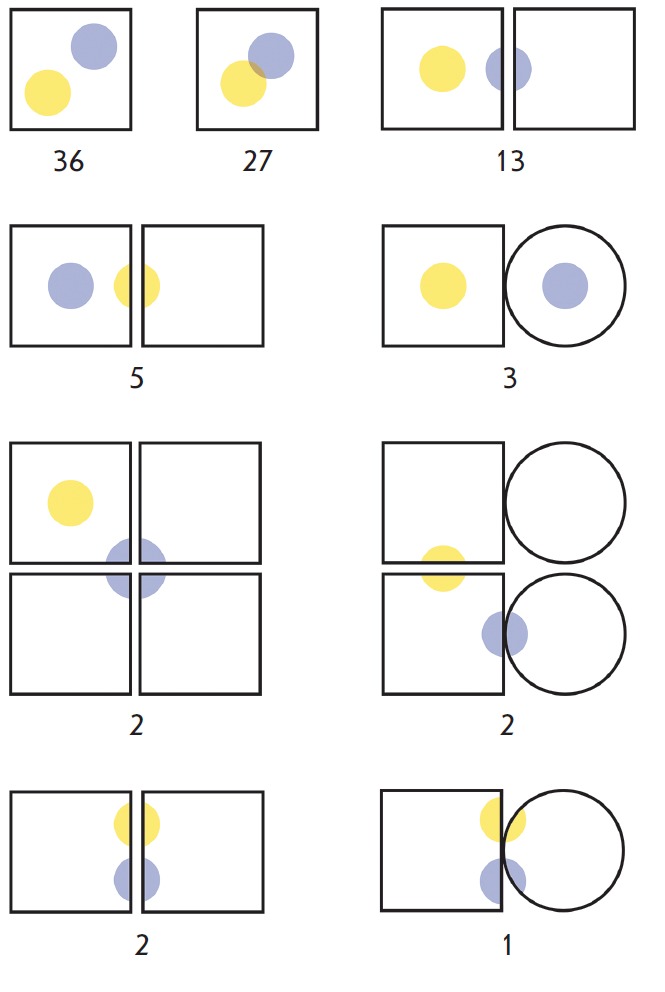
The ...

The CASBench benchmarking set contains 91 entries of enzymes with annotated catalytic and allosteric binding sites based on the information retrieved from the ASD and CSA databases and the results of the analysis of all crystallographic complexes with ligands in the respective sites. The CASBench includes enzymes of all major functional EC classes, which are presented in proportion to their occurrence in other databases (*Fig. 1*). Topological analysis showed that the CASBench contains proteins with different three-dimensional organizations; the catalytic and allosteric sites can be arranged in a variety of ways relative to each other (*Fig. 2*). In 70% of cases, the annotations describe monomeric proteins consisting of only one chain, and 30% of the entries correspond to multi-chain proteins consisting of several identical or unique subunits. In 5% of entries, both sites are formed within the intersubunit contact; in 22% of cases, only one site is located between the subunits and 73% of entries correspond to both sites being formed within the subunits. In all the CASBench annotations, different sites are topologically independent from each other (i.e., they are represented by separate cavities in the enzyme structure). In 30% of cases, the catalytic and allosteric sites either overlap or share a common border; in 70% of entries, both sites reside at a considerable distance from each other and do not overlap within the structure.

**Fig. 3:**
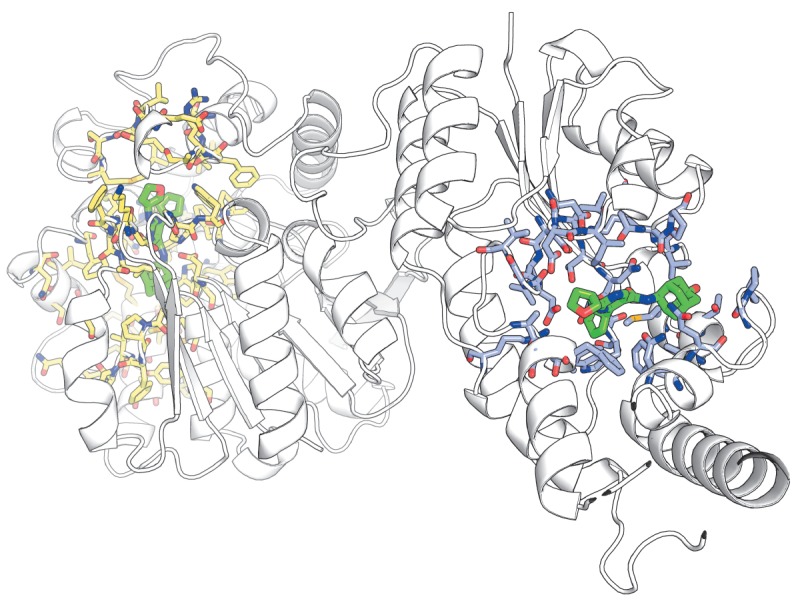
The ...

A CASBench entry for each listed protein has an identifier written as CAS0001 (CAS0002, CAS0003, etc.) and contains annotation of all sites, as well as associated ligands in all available crystallographic structures from the PDB database. The information is available as binary files in the PSE format for the PyMOL Molecular Graphics System (which can be used for visual expert analysis) and text files intended for automated processing. It should be noted that the original annotations of catalytic and allosteric sites in the ASD and CSA databases can consist of only several residues whose role in the protein function and regulation was verified experimentally. An important feature of the CASBench dataset is that the annotations of sites from the ASD and CSA databases are refined using the information from the crystallographic complexes of proteins with ligands. All residues directly interacting with ligands are shown in the benchmark set, which seems more convenient for further analysis and provides clear understanding of the size and boundaries of the binding sites (*Fig. 3*). For each CASBench entry, multiple alignments of a representative set of a corresponding protein family are also available in the FASTA format, which can be useful when testing algorithms that employ bioinformatic analysis to search for and/or rank ligand binding sites in protein structures (e.g., pocketZebra [13] and the like). All the data available in the CASBench can be operated offline on a local computer or online using built-in interactive tools. The web version of CASBench can be browsed via a single list of all available entries, or by searching by the protein PDB ID or keywords contained in the PDB annotation. Each CASBench entry is presented on a separate web page that contains information on all available PDB structures of the corresponding protein, annotated sites, and associated ligands. The annotated catalytic and allosteric sites can be visualized on the 3D structure or amino acid sequence of the selected protein using the built-in interactive tools (JSMol [50] and Strap [51], respectively). Online interactivity is implemented in HTML5 and requires neither plug-ins nor Java on the user’s computer.

## CONCLUSIONS

The trend in recent years has been increasing attention to allosteric regulation of the functional properties of proteins/enzymes and the search for complementary modulators as prototypes of novel drugs with lower toxicity thanks to a higher binding selectivity. Despite the growing interest in studying the relationship between the structure, function, and regulation, and in elaborating methods to search for new regulatory sites in protein structures, many questions are still to be answered. Therefore, further research into the field is required. In this paper, the CASBench benchmarking set was proposed, containing all enzymes with the catalytic and allosteric sites in their structures experimentally annotated based on the ASD, CSA, and PDB databases. The obtained set can be used to evaluate the efficiency of the existing methods and to develop/train prospective algorithms to search for new sites in protein structures, as well as to study the mechanisms of allosteric communication between topologically independent sites in a large collection of enzyme families. Establishing a relationship between structure, function, and regulation is expected to improve our understanding of the mechanisms of action of enzymes and open up new prospects for creating new drugs and designing more efficient biocatalysts.

## Abbreviations

ASD: Allosteric Database
CASBench: Catalytic and Allosteric Sites Benchmark set
CSA: Catalytic Site Atlas
HTML5: HyperText Markup Language version 5
PDB: Protein Data Bank
PSE: Pymol Session (binary file format)
